# Supplementary material for: Cryo-EM reveals species-specific components within the Helicobacter pylori Cag type IV secretion system core complex
Source: eLife. 2020 Sep 2;9:e59495. doi: 10.7554/eLife.59495 (PMC7511236; doi:10.7554/eLife.59495)
Supplement: Transparent reporting form [file elife-59495-transrepform.docx]

***eLife’s* transparent reporting form**

We encourage authors to provide detailed information *within their submission* to facilitate the interpretation and replication of experiments. Authors can upload supporting documentation to indicate the use of appropriate reporting guidelines for health-related research (see [EQUATOR Network](http://www.equator-network.org/%20)), life science research (see the [BioSharing Information Resource](https://biosharing.org/)), or the [ARRIVE guidelines](http://www.plosbiology.org/article/info:doi/10.1371/journal.pbio.1000412) for reporting work involving animal research. Where applicable, authors should refer to any relevant reporting standards documents in this form.

If you have any questions, please consult our Journal Policies and/or contact us: [editorial@elifesciences.org](mailto:editorial@elifesciences.org).

**Sample-size estimation**

- You should state whether an appropriate sample size was computed when the study was being designed
- You should state the statistical method of sample size computation and any required assumptions
- If no explicit power analysis was used, you should describe how you decided what sample (replicate) size (number) to use

Please outline where this information can be found within the submission (e.g., sections or figure legends), or explain why this information doesn’t apply to your submission:

Our sample size was determined by the number of high quality images we were able to collect on the Titan Krios with K2 Summit Direct Electron Detector. Information about sample size and how the data were collected is found in the Methods Section, Figure 1- Supplement Figure 1, and Supplementary Table 1.

**Replicates**

- You should report how often each experiment was performed
- You should include a definition of biological versus technical replication
- The data obtained should be provided and sufficient information should be provided to indicate the number of independent biological and/or technical replicates
- If you encountered any outliers, you should describe how these were handled
- Criteria for exclusion/inclusion of data should be clearly stated
- High-throughput sequence data should be uploaded before submission, with a private link for reviewers provided (these are available from both GEO and ArrayExpress)

Please outline where this information can be found within the submission (e.g., sections or figure legends), or explain why this information doesn’t apply to your submission:

The structure was determined using established cryo-EM image processing and validation methods.

**Statistical reporting**

- Statistical analysis methods should be described and justified
- Raw data should be presented in figures whenever informative to do so (typically when N per group is less than 10)
- For each experiment, you should identify the statistical tests used, exact values of N, definitions of center, methods of multiple test correction, and dispersion and precision measures (e.g., mean, median, SD, SEM, confidence intervals; and, for the major substantive results, a measure of effect size (e.g., Pearson's r, Cohen's d)
- Report exact p-values wherever possible alongside the summary statistics and 95% confidence intervals. These should be reported for all key questions and not only when the p-value is less than 0.05.

Please outline where this information can be found within the submission (e.g., sections or figure legends), or explain why this information doesn’t apply to your submission:

This manuscript reports the structure of the *H. pylori* Cag T4SS determined using single particle cryo-EM. No statistical reporting was required for these studies. We did use the Gold-Standard FSC criteria to determine the resolution of our cryo-EM model. We also validated the models built from the cryo-EM map using Phenix, Molprobity scores, Clashscores and Ramachandran plots. This information can be found in Supplemental Table 1.

(For large datasets, or papers with a very large number of statistical tests, you may upload a single table file with tests, Ns, etc., with reference to sections in the manuscript.)

**Group allocation**

- Indicate how samples were allocated into experimental groups (in the case of clinical studies, please specify allocation to treatment method); if randomization was used, please also state if restricted randomization was applied
- Indicate if masking was used during group allocation, data collection and/or data analysis

Please outline where this information can be found within the submission (e.g., sections or figure legends), or explain why this information doesn’t apply to your submission:

Particles in our data set were sorted by 2D and 3D averaging during data processing. We used focused refinement to improve the resolution of sub-complexes in the T4SS. The summary of this process and how these decisions were made are described in the methods section and shown in Figure 1- Supplement Fig. 1.

**Additional data files (“source data”)**

- We encourage you to upload relevant additional data files, such as numerical data that are represented as a graph in a figure, or as a summary table
- Where provided, these should be in the most useful format, and they can be uploaded as “Source data” files linked to a main figure or table
- Include model definition files including the full list of parameters used
- Include code used for data analysis (e.g., R, MatLab)
- Avoid stating that data files are “available upon request”

Please indicate the figures or tables for which source data files have been provided:

All cryo-EM data included in this manuscript are available through the Electron Microscopy Data Bank (EMD-20021, EMD-22081, EMD-22076, and EMD-22077). All models that were constructed from these data are available via the Protein Data Bank (PDB 6X6S, 6X6J, 6X6K, and 6X6L) and model validation reports are included with the submission. Supplementary Table 1 provides a summary of the datasets and models and Fig. 1-Supplement Figure 1 provides the workflow for image processing.
